# A Numbers Game: Ribosome Densities, Bacterial Growth, and Antibiotic-Mediated Stasis and Death

**DOI:** 10.1128/mBio.02253-16

**Published:** 2017-02-07

**Authors:** Bruce R. Levin, Ingrid C. McCall, Véronique Perrot, Howard Weiss, Armen Ovesepian, Fernando Baquero

**Affiliations:** a Department of Biology, Emory University, Atlanta, Georgia, USA; b Department of Mathematics, Georgia Institute of Technology, Atlanta, Georgia, USA; c Department of Veterinary Disease Biology, University of Copenhagen, Copenhagen, Denmark; d Ramón y Cajal Institute for Health Research (IRYCIS), Ramón y Cajal University Hospital, CIBERESP, Madrid, Spain; Indiana University Bloomington

## Abstract

We postulate that the inhibition of growth and low rates of mortality of bacteria exposed to ribosome-binding antibiotics deemed bacteriostatic can be attributed almost uniquely to these drugs reducing the number of ribosomes contributing to protein synthesis, i.e., the number of effective ribosomes. We tested this hypothesis with *Escherichia coli* K-12 MG1655 and constructs that had been deleted for 1 to 6 of the 7 rRNA (*rrn*) operons. In the absence of antibiotics, constructs with fewer *rrn* operons have lower maximum growth rates and longer lag phases than those with more ribosomal operons. In the presence of the ribosome-binding “bacteriostatic” antibiotics tetracycline, chloramphenicol, and azithromycin, *E. coli* strains with 1 and 2 *rrn* operons are killed at a substantially higher rate than those with more *rrn* operons. This increase in the susceptibility of *E. coli* with fewer *rrn* operons to killing by ribosome-targeting bacteriostatic antibiotics is not reflected in their greater sensitivity to killing by the bactericidal antibiotic ciprofloxacin, which does not target ribosomes, but also to killing by gentamicin, which does. Finally, when such strains are exposed to these ribosome-targeting bacteriostatic antibiotics, the time before these bacteria start to grow again when the drugs are removed, referred to as the post-antibiotic effect (PAE), is markedly greater for constructs with fewer *rrn* operons than for those with more *rrn* operons. We interpret the results of these other experiments reported here as support for the hypothesis that the reduction in the effective number of ribosomes due to binding to these structures provides a sufficient explanation for the action of bacteriostatic antibiotics that target these structures.

## IMPORTANCE

Chemotherapeutic agents, including antibiotics, have been used for more than a century; nevertheless, there are still major gaps in our understanding of how these drugs operate which limit future advances in antibacterial chemotherapy. Although the molecular mechanisms by which antibiotics bind to their target structures are largely known, fundamental questions about how these drugs actually kill and/or inhibit the replication of bacteria remain unanswered and subjects of controversy. We postulate that for the broad class of ribosome-binding bacteriostatic antibiotics, their reducing the number of active (functional) ribosomes per cell provides a sufficient explanation for the abatement of replication and the low rate of decline in densities of viable cells of bacteria exposed to these drugs. Using *E. coli* K-12 constructs with deletions of from one to six of the seven ribosome-RNA operons and the ribosome-binding bacteriostatic antibiotics tetracycline, chloramphenicol, and azithromycin, we tested this hypothesis. The results of our experiments are consistent with this “numbers game” hypothesis.

## INTRODUCTION

Antimicrobial chemotherapeutic agents, as we now know them, have been studied for more than a century, since Paul Ehrlich developed arsphenamine, also known as Salvarsan or compound 606, an organoarsenic drug introduced at the beginning of the 1910s for the treatment of *Treponema pallidum* infections (syphilis) (1). In the course of this time, many different naturally occurring, synthetic and semisynthetic antibiotics have been developed and used. For virtually all of these drugs, the molecular structure and target of action and the molecular basis of the interactions with the target have been elucidated. The literature abounds with colorful three-dimensional (3D) diagrams of antibiotics binding to and modifying the structure of their target molecules (see, for example, references 2, 3, and 4).

Indeed, from reviews of the antibiotic and antibiotic treatment literature, one may get the impression that, for the vast majority of antibiotics currently employed, we know virtually all that is meaningful. Arguably, but far from surely, that may well be the case for the use of these drugs clinically. On the other hand, at a mechanistic level there are fundamental unanswered questions, such as how bactericidal antibiotics actually kill bacteria. The controversy about the role of reactive oxygen species (ROS) in the killing of bacteria by bactericidal antibiotics (5–8) (also see reference 9 for a commentary) is a testimony to the existing knowledge gaps about these mechanisms. One would assume that, after more than a century of studying antibiotics, there would be a widely accepted answer(s) to this fundamental question.

Unanswered questions about how antibiotics actually work are not restricted to the mechanism by which they kill bacteria. Many of the major antibiotic drugs act primarily by inhibiting the replication of the bacteria and are deemed “bacteriostatic” rather than “bactericidal.” The majority of bacteriostatic antibiotics employed, including agents such as chloramphenicol, the tetracyclines, the macrolides (as erythromycin), and the oxazolidinones (as linezolid), target ribosomes. How these drugs bind to ribosomes, the binding sites, the kinetics of their associations with these structures (binding rates), and their effects on protein synthesis and the metabolic rates of bacteria have been extensively studied (e.g., see references 10, 11, 12, 13, 14, and 15). Nevertheless, it remains unclear why these ribosome-targeting antibiotics are bacteriostatic and why their use at low concentrations reduces the growth rates of bacterial populations and at higher concentrations prevents the growth of and leads to low rates of decline in the viable cell densities of these populations.

In this report, we present (“venture” may prove a more prudent verb choice) a general hypothesis that can account for these properties of ribosome-targeting bacteriostatic antibiotics. Using constructs of *Escherichia coli* with different numbers of rRNA (*rrn*) operons and bacteriostatic and bactericidal antibiotics of different classes, we tested this hypothesis. We interpret the results of our experiments as support for this hypothesis.

## RESULTS

### A hypothesis for the bacteriostatic and bactericidal activity of ribosome-binding antibiotics.

**FIG 1:**
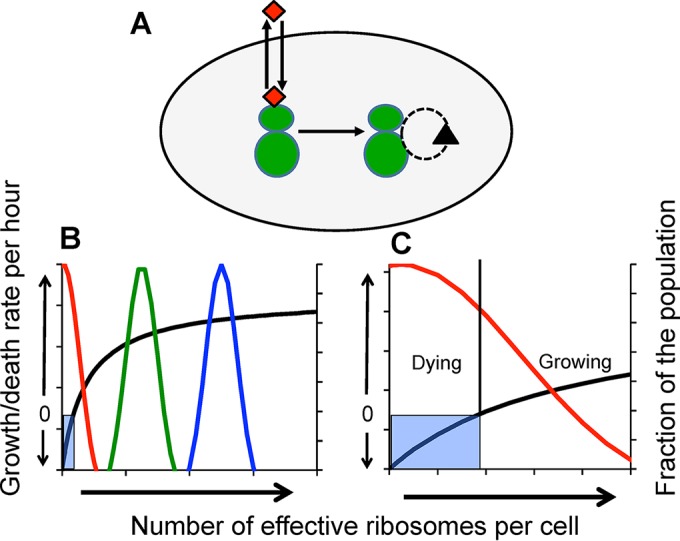
A model for the numbers game hypothesis for the action of ribosome-binding bacteriostatic antibiotics. (A) Turnover of ribosome (the green “shmoos”). The dashed circle with an arrow represents degradation and replacement (biogenesis) of ribosomes in a cell. The red diamonds represent antibiotics entering the cells and binding to and being released from the ribosomes. (B) Growth rate and death rate as functions of the number of ribosomes (the black line) (a Hill function; see the text). When the number of ribosomes is in the shaded area, cells die at a low rate. The blue, green, and red lines are normal distributions for the fraction of the population with the corresponding number of functional (not bound to drug) ribosomes. (C) Blow-up, growth, and death when the average number of ribosomes is low.

Our hypothesis for the mechanisms of action of ribosome-binding bacteriostatic antibiotics is founded on the classical studies of Kjeldgaard and Kurland (16), Ecker and Schaechter (17), and Davis and colleagues (18) demonstrating that the rates of growth of bacteria are directly proportional to the number of ribosomes in a cell. Central to this hypothesis is the assumption that there is a distribution of ribosome numbers among the members of a population of bacteria (19, 20). In the absence of antibiotics and in the presence of sufficient nutrients, the population grows at a rate that depends on the average number of ribosomes contributing to protein synthesis among its members. In Fig. 1, we illustrate this hypothesis.

The model depicted in Fig. 1A is based on that by Greulich and colleagues (21). There is a continuous turnover of ribosomes in a cell. The bacteria take up the antibiotic, which binds to the ribosomes and thereby removes them from the pool of ribosomes contributing to protein synthesis. As a consequence of this encounter with these bacteriostatic antibiotics, the average number of “effective ribosomes” borne by members of the population is reduced. At some rate, the antibiotics dissociate and the previously drug-bound ribosome becomes effective again (22). We assume a Hill function for the relationship between the number of ribosomes borne by a cell and its growth rate (shown as the black line in Fig. 1B and C) as follows:

$$\psi (r)=(\psi_{MAX}-\psi_{MIN})\frac{r}{\left(r+\text{κ}\right)}+\psi_{MIN}$$

where ψ_*MAX*_ (> 0) per cell per unit time is the maximum growth rate, ψ_*MIN*_ (< 0) is the death rate, κ is the Hill (shape) parameter, and *r* is the number of ribosomes in a cell.

In the absence of the antibiotic and in the presence of nutrients, the vast majority of the population has a sufficient number of ribosomes to replicate and the population grows (the blue line in Fig. 1B). If the average number of effective ribosomes is reduced (the green distribution in Fig. 1B), the population can still replicate but does so at a rate lower than that which obtains with a higher average number of ribosomes (Fig. 1B). By further lowering the number of effective ribosomes which is anticipated due to binding by bacteriostatic antibiotics, the net growth rate of the population at large becomes negative (the red line in Fig. 1B, and Fig. 1C). Depending on their number of effective ribosomes, individual cells would either be dividing or dying. In Fig. S1 in the supplemental material, we use a stochastic simulation for a numerical illustration of the relationship between the distribution of ribosome numbers among cells and the growth/death rate of the population ψ_(*r*)_ as depicted in Fig. 1B and C.

10.1128/mBio.02253-16.1 FIG S1  A stochastic model for the numbers game hypothesis for the action of ribosome-binding bacteriostatic antibiotics, stochastic simulation. We simulate a population consisting of *n* = 1e6 cells, where for each cell the number of ribosomes is an independent normal random variable with standard deviation 0.1* *m*, where *m* denotes the mean. We assume that a cell contributes positively to the population’s exponential growth rate if it contains at least a threshold number of ribosomes and negatively if it contains fewer and that the cell’s contribution increases with increasing numbers of ribosomes and saturates. We model this using the function ψ(*r*) = (ψ_*MAX*_ − ψ_*MIN*_) [*r*/(*r* + l)] + ψ_*MIN*_ to represent the exponential growth/death rate of a cell having *r* ribosomes. For each cell, we apply the function ψ to the randomly chosen number of ribosomes to obtain the distribution of individual exponential growth/death rates. The average *m* value distributions are indicated as follows: red, *m* = 1,000; green, *m* = 10,000; olive/yellow, *m* = 30,000; blue, *m* = 50,000. In the distribution for *m* = 1e4, the majority of cells are dying (*y* < 0) but some are growing (*y* > 0). The distributions with *m* = 30,000 and *m* = 50,000 have small variances, since, in these ranges, the curve for the ψ function is almost flat. The Mathematica notebook for this simulation is available at http://www.eclf.net. Download FIG S1, TIF file, 0.5 MB. Copyright © 2017 Levin et al. 2017 Levin et al. This content is distributed under the terms of the Creative Commons Attribution 4.0 International license.

### Predictions.

There are three testable (and rejectable) predictions:

1. A reduction in the average number of ribosomes would be made manifest by a lower maximum growth rate for the population at large.
2. When the average number of ribosomes is further reduced, ribosome-binding bacteriostatic antibiotics become increasingly bactericidal.
3. Because of the binding of the antibiotic to and dissociation of the antibiotic from the ribosomes, the extent to which bacteriostatic antibiotics reduce the growth rate and kill would be proportional to the concentration of the drug.

If we further assume that (i) the transition from non-growth to growth when populations are provided with fresh media requires the production of a specific number of new, functional ribosomes and (ii) the time required to achieve that number is inversely proportional to the average number of functional ribosomes borne by members of the population in that static state, there are two additional predictions:

1. In the absence of antibiotics, a reduction in the average number of ribosomes would be manifest by an increase in the length of time before a stationary-phase population starts to grow when fresh resources are made available (the lag phase).
2. When stasis is due to the presence of ribosome-binding antibiotics, a reduction in the average number of functional ribosomes would be reflected as an increase in the time before the population grows after the antibiotics are removed and fresh resources are made available (the post-antibiotic effect [PAE]) (23).

### Ribosome operon deletion strains and numbers of ribosomes.

To test the validity of these predictions and thereby the ribosome number hypothesis for the action of ribosome-binding bacteriostatic antibiotics, we used a set of *E. coli* K-12 strains constructed by S. Quan and colleagues (24, 25) with deletions of from 1 to 6 of the 7 *E. coli* rRNA (*rrn*) operons (designated D1 to D6) and the *E. coli* MG1655 strain (designated MG) from whence they were derived. In an effort to determine the relationship between the number of ribosome (*rrn*) operons and the number of ribosomes, we used a Bioanalyzer to estimate the relative amounts of 16S and 23S rRNA per unit of total cell RNA of these deletion strains. The results of this analysis for three independent RNA extractions from 24-h stationary-phase cultures of MG1655 and the 6 *rrn* operon deletion strains are depicted in Fig. S2.

10.1128/mBio.02253-16.2 FIG S2  Bioanalyzer results: percentages of total area of 16S and 23S RNA for *E. coli* constructs with from 1 to 6 rRNA *rrn* operons. Results of three independent RNA preparations and analyses, one with red lines and ticks and two with blue lines and ticks, are shown. Linear regression and significance-level one-tail *t* test results are shown. Download FIG S2, TIF file, 0.5 MB. Copyright © 2017 Levin et al. 2017 Levin et al. This content is distributed under the terms of the Creative Commons Attribution 4.0 International license.

We interpret the results of this analysis to be consistent with the hypothesis that, under the conditions of these experiments (*E. coli* grown at 37°C in glucose minimal medium supplemented with 0.2% casein amino acids), the number of ribosomes is proportional to the number of ribosomal operons; constructs with fewer *rrn* operons have fewer ribosomes. There is a significant increase in the fraction of 16S rRNA and 23S rRNA with increasing numbers of *rrn* operons. It should be noted, however, that a linear relationship between the number of *rrn* operons and the amount of rRNA is not expected, because of the higher expression of the remaining *rrn* copies in *rrn* operon-deleted mutants (26).

### Ribosome numbers and rates of growth and time of lag.

In accord with the hypothesis depicted in Fig. 1 and the predictions based on this model, when the average number of ribosomes is sufficiently high, the rate of growth of the bacteria is predicted to be relatively independent of that number. When the number of ribosomes is further reduced, the rate of growth of the bacteria is predicted to decline and become increasing proportional to the number of ribosomes. The results of studies estimating the growth rates of *E. coli* in lysogeny broth (LB) (24, 27) support these predictions.

**FIG 2:**
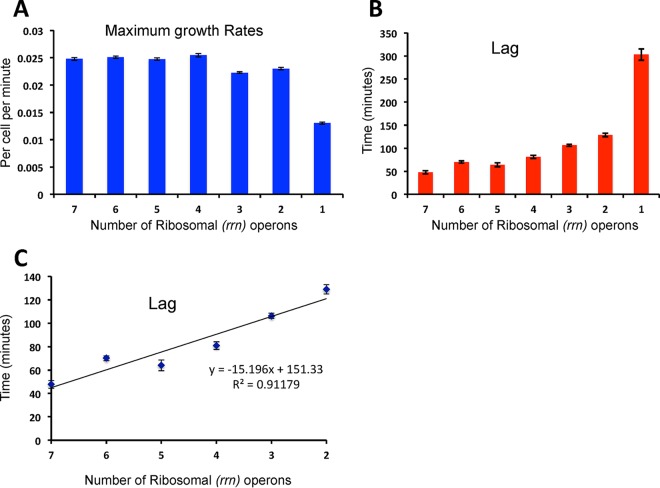
Rates of growth and lengths of lags for *E. coli* as a function of numbers of ribosomal (*rrn*) operons estimated from changes in optical density (600 nm Bioscreen) and CFU data. (A) Means and standard errors of the maximum growth rates (the Malthusian parameter [MP]). (B) Means and standard errors of the length of the lag. (C) Functional relationship between the length of the lag phase and the number of ribosomal operons for MG1655 and constructs with 2 to 6 *rrn* operons. Data were compiled from separate Bioscreen runs with a minimum of 20 points for each strain.

To obtain a broader perspective on the effects of deletions of ribosomal operons (which we use as a proxy for the average number of ribosomes) on the dynamics of growth of *E. coli* and on the robustness and generality of the results reported in references 24 and 27, we used a Bioscreen automated plate reader to follow the changes in the optical densities of growing populations of *E. coli* for 20 h or more. The bacteria were incubated at 37°C and continuously shaken, with optical densities (ODs) at 600 nm estimated every 5 min. In these experiments, we used the medium (glucose minimal medium supplemented with 0.2% casein amino acids) employed for the 16S-23S RNA assay whose results are presented in Fig. S2 and in the experiments that follow. Estimates of the maximum growth rates (the Malthusian parameters [MP]) and the lengths of lags as functions of the number of *rrn* operons are depicted in Fig. 2.

There was no apparent effect on the maximum growth rate, MP, when the number of *rrn* operons was 4 or greater [F(3,121) = 1.56] (*P* = 0.203). The MP values of the constructs with fewer than 3 *rrn* operons were substantially lower than the MP values of those with more [F(2,92) = 45.32] (*P* < 0.0001). The apparent greater MP of the constructs with 2 *rrn* operons than of those with 3 [F(1,63) = 6.9] is statistically significant (*P* = 0.011), but the difference is negligible. The most profound effect of ribosome operon numbers on maximum growth rate is seen for the construct with only 1 *rrn* operon, for which the value is only 57 percent as great as the value for those with 2 *rrn* operons.

The length of the lag declines with the number of ribosomal operons (Fig. 2B). For the constructs with from 2 to 7 *rrn* operons, the relationship between the lag and the number of *rrn* operons is roughly linear, with a highly significant slope (*P* < 0.00010), for 188 degrees of freedom: *P*(−17.0 < ρ < −13.6) = 0.95. The length of the lag phase increased precipitously for the construct with 1 *rrn* operon.

### Ribosome numbers and the bactericidal effects of bacteriostatic antibiotics.

In accord with the ribosome number hypothesis for the static and cidal activity of bacteriostatic antibiotics that bind to these structures, the expression of a critical number of vital proteins is needed to sustain cellular life. Consequently, if the average number of ribosomes contributing to protein synthesis is reduced, these drugs should become increasingly bactericidal. To test this hypothesis, we exposed MG1655 and the constructs with deletions of from 1 to 6 *rrn* operons to 25, 125, and 40 µg/ml of tetracycline (TET), chloramphenicol (CAM), and azithromycin (AZI), respectively. As our measure of the susceptibility of these strains to killing by these drugs, we used the hourly rate of growth/death, ψ, calculated from the viable cell densities (CFU data) of growing cultures immediately before exposure to the antibiotics, *N*(0), and after 24 h of exposure, *N*(24), as follows: $\psi_{i}\;=\;\frac{1}{24}\;\;\ln\;\;\left[\frac{N(24)}{N(0)}\right]$. The estimates of *N*(0) and *N*(24) for these calculations were the mean values estimated from three (and occasionally two) separate serial dilutions and platings.

**FIG 3:**
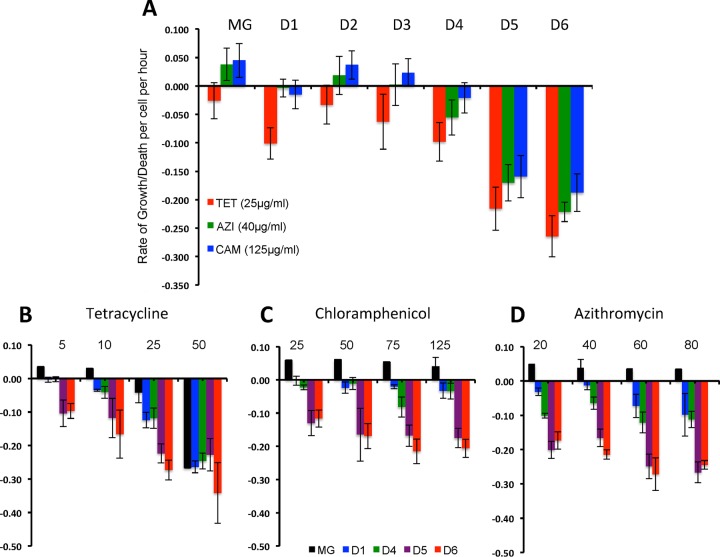
Susceptibility to killing by ribosome-targeting bacteriostatic antibiotics. (A) Hourly growth/death rates for exponentially growing MG1655 (MG) and constructs with from 0 to 6 ribosomal *rrn* operon deletions (MG and D1 to D6) exposed to 25, 125, and 40 µg/ml of tetracycline (TET), chloramphenicol (CAM), and azithromycin (AZI), respectively. (B to D) Hourly growth/death rates of *E. coli* MG1655 and constructs with 7, 6, 3, 2, and 1 *rrn* operons (respectively, MG, D1, D4, D5, and D6) exposed to different concentrations of tetracycline, chloramphenicol, and azithromycin before the growing cultures were exposed to the drugs and after 24 h of exposure. The results shown in panel A represent the means and standard errors of the estimates of ψ for from 7 to 10 independent experiments of this growth/death rate parameter, and those shown in panel B represent the means and standard errors of the estimates of ψ for from 2 to 4 independent experiments.

The results of a compendium of from 7 to 10 of these time-kill experiments are presented in Fig. 3A. As measured by the rate of kill of these antibiotics, ψ, constructs with only 1 and 2 *rrn* operons are considerably more susceptible to killing by these drugs than those with more *rrn* operons. The concentrations of antibiotics used in the experiments whose results are presented in Fig. 3A were chosen because they were substantially above the MICs of these antibiotics for these *E. coli* constructs (Table S1) and for susceptible *E. coli* as a species (28). In Fig. 3B, we present estimates of ψ for a compendium of from 2 to 4 time-kill experiments run with different concentrations of these “bacteriostatic” antibiotics. These experiments provide evidence that the constructs with 1 and 2 ribosomal operons, D5 and D6, are more susceptible to killing by these drugs than those with more *rrn* operons. As expected, these results also indicate that the rate of killing by these “bacteriostatic” antibiotics and particularly by tetracycline increases with the concentration of the drug (29).

10.1128/mBio.02253-16.4 TABLE S1  MICs (in micrograms per milliliter) of the antibiotics used these experiments for MG1655 and the *rrn* operon deletion strains. Download TABLE S1, DOCX file, 0.01 MB. Copyright © 2017 Levin et al. 2017 Levin et al. This content is distributed under the terms of the Creative Commons Attribution 4.0 International license.

### Ribosome numbers and the susceptibility of *E. coli* to bactericidal antibiotics.

The preceding results support the hypothesis that reductions in ribosome numbers make *E. coli* more susceptible to killing by the ribosome-targeting bacteriostatic antibiotics (tetracycline, chloramphenicol, and azithromycin) and that this bactericidal effect is proportional to the concentration of the antibiotic. There is, however, a caveat that has to be addressed: to wit, that reductions in ribosome numbers make *E. coli* more susceptible to antibiotic-mediated killing for reasons other than the numbers of ribosomes. In accord with this caveat, antibiotics that do not act by binding to ribosomes would also be more bactericidal when the average number of ribosomes is reduced. To test this, we performed time-kill experiments similar to those described above using the bactericidal antibiotic ciprofloxacin, which acts on DNA synthesis (30) rather than ribosomes, and also gentamicin, which does target ribosomes (31).

**FIG 4:**
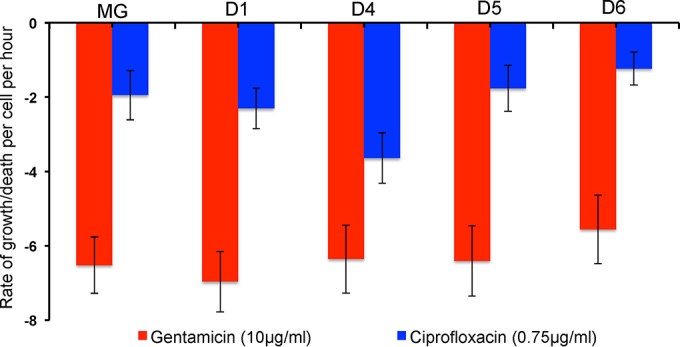
Hourly rates of decline in the viable cell density of growing cultures of MG1655 and constructs with 6, 3, 2, and 1 *rrn* operons (respectively, D1, D4, D5, and D6) exposed to 10 µg/ml gentamicin and 0.75 µg/ml ciprofloxacin. Data represent means and standard errors of the kill rates (ψ*i*) determined for 2 or 3 separate experiments.

The results of this experiment are presented in Fig. 4. This experiment suggests that there is either no relationship between the susceptibility of the strains with fewer ribosomal operons and killing by these bactericidal antibiotics or, possibly, lower sensitivity of the construct with 1 ribosomal operon. See the Discussion for a consideration of why this might be the case.

### Postantibiotic effects.

The increase in the lag with increases in the number of *rrn* operon deletions (reductions in the number of ribosomes) (Fig. 2) is also what would be anticipated from the model depicted in Fig. 1. To come out of stationary phase, the bacteria have to produce a sufficient number of functional ribosomes to replicate. In this interpretation, those that had fewer ribosomal operons and would thereby start with lower numbers of ribosomes would take longer to reach the threshold number of ribosomes required for replication. Exposure to antibiotics also increases lags. Although the resources needed for replication abound and the bacteria are not at stationary phase, there is a period of time after antibiotics are removed before the population starts to grow. One interpretation of the increased lag following exposure to these drugs, i.e., of this post-antibiotic effect (PAE) (23), for ribosome-binding bacteriostatic antibiotics is that as a consequence of binding to these structures, the average number of effective ribosomes is reduced and more unbound ribosomes have to be produced before the population grows compared to a corresponding population not exposed to these drugs (32, 33). In this interpretation, reductions in the average number of ribosomes would further augment the length of the PAE.

**FIG 5:**
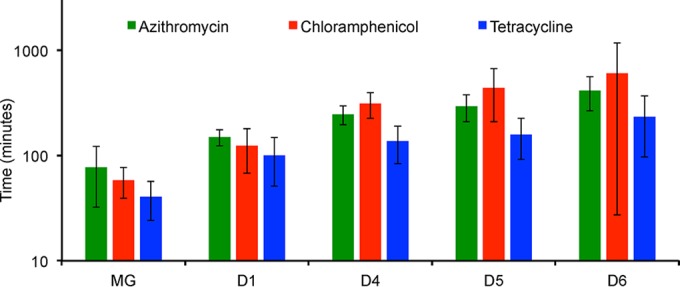
Post-antibiotic effect (PAE) for MG1655 (MG) and constructs with 6, 3, 2, and 1 *rrn* operons (D1, D4, D5, and D6, respectively) exposed to 25, 125, and 40 µg/ml of tetracycline (TET), chloramphenicol (CAM), and azithromycin (AZI), respectively. Data represent differences between the estimated lag values determined following exposure to the antibiotics and those observed in the absence of these drugs. Means and standard errors of PAEs for three separate experiments are shown.

To test this hypothesis, we estimated the length of the PAE for the ribosome-targeting bacteriostatic antibiotics considered here for MG1655 and *E. coli* constructs deleted for 1, 4, 5, and 6 *rrn* operons (D1, D4, D5, and D6, respectively). The details of the protocol are presented in Materials and Methods. The results of a compendium of three of these PAE experiments are presented in Fig. 5.

As suggested by the magnitude of the standard errors, there was considerable variation among the results of the experiments in the estimates of the PAEs and particularly that for chloramphenicol and strain D6 with 1 *rrn* operon. Be that as it may, these results certainly suggest that the lengths of the PAE for the constructs with fewer *rrn* operons, D4, D5, and D6, are greater than the lengths of the PAE for those with more *rrn* operons, MG and D1.

## DISCUSSION

By binding to these macromolecular complexes, ribosome-targeting bacteriostatic antibiotics reduce the number of ribosomes contributing to protein synthesis. We postulate that this reduction in the average number of effective ribosomes is a sufficient explanation for the abatement of growth (stasis) and the low rate of decline in the viable cell densities of populations of bacteria exposed to ribosome-targeting bacteriostatic antibiotics. By the word “sufficient” we are proposing that the mode of action of these antibiotics is essentially a ribosome “numbers game” and that it is not necessary to assume that other processes are involved.

We interpret the results of our experiments performed with *E. coli* MG1655 and constructs deleted for 1 to 6 of the 7 ribosomal operons as evidence in support of this numbers game hypothesis for the action of ribosome-targeting bacteriostatic antibiotics. As anticipated by this hypothesis and observed in our experiments, three predictions have been met. (i) When exponentially growing populations of *E. coli* are exposed to supra-MICs of the ribosome-targeting bacteriostatic antibiotics tetracycline, chloramphenicol, and azithromycin, the rate at which constructs with 1 and 2 ribosomal operons are killed is substantially greater than that for those with more *rrn* operons. (ii) This greater sensitivity of constructs with 1 and 2 *rrn* operons to killing by bacteriostatic antibiotics is not manifest by their being more susceptible to killing by ciprofloxacin, which does not target ribosomes. (iii) The length of the lag following removal of the antibiotic (the post-antibiotic effect [PAE]) is greater for constructs with only 1 or 2 *rrn* operons than for constructs with more *rrn* operons.

Central to this interpretation of our experiments is the proposition that the average number of ribosomes borne by cells is proportional to the number of ribosomal RNA operons. The observation that the fraction of 16S RNAs and 23S RNAs declines in proportion to the number of *rrn* operon deletions is consistent with this proposition. Also consistent are the growth dynamic data presented in this report (Fig. 2) and in reference 24, along with classical studies of the rates of growth of *E. coli* and the numbers of ribosomes reported by Kjeldgaard and Kurland (16), Ecker and Schaechter (17), and Davis and colleagues (18).

On first consideration, it may seem that the observation that constructs with 1 and 2 ribosomal operons are not killed by the ribosome-targeting, bactericidal antibiotic gentamicin at a rate different from the rate seen with those with more ribosomal operons (Fig. 4), may seem inconsistent with this numbers game hypothesis. We suggest this is not the case. Even if there were a contribution of ribosome numbers to the rate of kill of the magnitude observed for the bacteriostatic antibiotics and constructs with 1 and 2 *rrn* operons, it would be hard to detect this effect for bacteria exposed to supra-MICs of gentamicin. The maximum rate of killing of a construct with 6 *rrn* operons by bacteriostatic antibiotics is on the order of −0.30 per h, which obtained at approximately 80×, 33×, and 32× MIC for tetracycline, chloramphenicol, and azithromycin, respectively. At approximately 4× MIC, the rate of gentamicin-mediated kill is on the order of −7.0 per h.

If anything, this difference in the kill rate of gentamicin relative to those of the ribosome-targeting bacteriostatic drugs supports the hypothesis that when bacteria are exposed to supra-MICs of this aminoglycoside, processes other than the reduction in the number of effective ribosomes, such as a “surface-cidal effect” (34, 35), are responsible for the high rate at which bacteria are killed by these drugs. The possibility of ribosomes producing an “binding sink effect” with respect to aminoglycosides, massively increasing drug uptake (energy-dependent phase II) and resulting in a collapse of proton motive force and, finally, lethal cell membrane damage, is certainly of interest (36, 37). Reductions in the number of ribosomes in strains with increasing numbers of *rrn* operon deletions will decrease the binding sink effect and thus aminoglycoside uptake, such that deletions would decrease rather than increase the rate at which supra-MIC concentrations of aminoglycosides kill bacteria. Similarly, as a consequence of having fewer ribosomes, we would anticipate a reduction in the absolute number of mistranslated and misfolded proteins and thus a lower rate of production of the toxic compounds that are considered to be responsible for killing bacteria exposed to gentamicin or other aminoglycosides (5, 38–40). Moreover, because the rate of replication would be reduced, there should be less misreading. For example, *rpsL* mutations increase the fidelity of protein synthesis by reducing the rates of protein elongation and thereby allowing for more error correction.

### Consistent and sufficient.

We interpret the results of these experiments to be consistent with and thereby provide support for the ribosome numbers game hypothesis for the mechanisms of action of ribosome-targeting bactericidal antibiotics. We recognize that there may be more to the association between *rrn* operon numbers and the susceptibility of bacteria to killing by ribosome-targeting bacteriostatic antibiotics and the length of the post-antibiotic lag (PAE) than simple reductions in the effective number of ribosomes as suggested by our model. Moreover, processes other than removing ribosomes from the effective pool by binding can account for these drugs reducing the number of ribosomes contributing to protein synthesis. Most prominent of these other mechanisms is that of binding to these macromolecules, these antibiotics also inhibit the biogenesis of new ribosomes (10, 11, 41). That is, as a consequence of reducing rates of protein synthesis, there is imbalance in the production of ribosomal components (42). With respect to our model, this would be another reason that bacteriostatic antibiotics that target these macromolecules would reduce their effective numbers.

It should be noted that the model depicted in Fig. 1 makes a prediction about the mode of action of ribosome-targeting bacteriostatic antibiotics that is independent of the ribosome number elements of this hypothesis. It predicts that when growing populations of bacteria are confronted with these drugs, a fraction of the population will be replicating at a low rate and another fraction will be dying. While this hypothesis cannot be tested with population-level data of the sort employed here, it can be tested and rejected by following the fate of individual cells in growing cultures exposed to ribosome-targeting antibiotics.

### Mechanisms and mechanisms and unanswered questions.

At one level, the postulated reduction in the average number of effective ribosomes represents a mechanism explaining how ribosome-targeting antibiotics arrest the growth, reduce the metabolic rate, and increase the rate of decline in viable cell density of bacteria exposed to these agents. However, as an explanation of the mode of action of ribosome-binding bacteriostatic antibiotics, the postulated “numbers game” is no more complete than mistranslation producing “toxic” compound explains how, when exposed to ribosome-targeting bactericidal antibiotics, bacteria are killed at a high rate; it is still necessary to account for how these “toxins” kill (5, 9). A comprehensive mechanistic explanation for the action of ribosome-targeting bacteriostatic drugs requires more quantitative information about the dynamics of ribosome degradation and production, the binding and release of the antibiotics, and the relationship between protein synthesis and other factors contributing to the rates of growth and death of bacteria.

It was classically proposed that, as temporary arrest of bacterial protein synthesis is not *per se* lethal, inhibitors of protein synthesis are bacteriostatic if they do not form irreversible bonds with the ribosome and that, if they do, they are bactericidal (43). Consistent with this interpretation and this ribosome numbers game hypothesis is the synergy between ribosome-binding antibiotics of different types (44). However, the validity of this statement about binding has not been fully demonstrated; the advent of ribosomal crystallography and nuclear magnetic resonance for the study of the interaction of antibiotics with the ribosome (45) has shown that macrolides that bond to the ribosome leave the complex very slowly, whereas aminoglycosides, being reversible binders, are more highly cidal drugs. Certainly, we need more data on the proteome of bacterial cells confronted with ribosome inhibitors to better address to this issue. As with a recent published, elegant, jointly theoretical and experimental study of the growth-dependent action of ribosome-targeting antibiotics (21), at this juncture the numbers game hypothesis presented here is arguably more phenomenological than fully mechanistic.

Be that as it may, the numbers game hypothesis presented in this study may have practical implications in chemotherapy. In contrast to what is generally assumed for antimicrobial agents and is certainly true for bactericidal drugs, bacteriostatic antibiotics might increase rather than decrease their efficacy in slowly growing bacterial populations. This hypothesis suggests that bacteriostatic drugs kill a greater fraction of the treated bacterial populations under certain cellular conditions that are expected to occur *in vivo*, such as a shortage of nutrients, than under *in vitro* conditions. Could there be sites of infection where, because of the distribution of ribosome numbers of the infecting bacteria, antibiotics that are considered bacteriostatic are bactericidal? Could this be the reason or part of the reason that bacteriostatic antibiotics targeting ribosomes are as effective clinically for treating acute infections as bactericidal drugs (46, 47)? Do the intracellular concentrations of such static agents as macrolides, tetracyclines, or chloramphenicol (in the range of those used in our experiments) influence the expected intracellular bacteria with lower ribosome numbers? Could the distribution of ribosome numbers be the reason why bacteriostatic antibiotics are effective (and are frequently the drugs of choice) for treating infections by mostly intracellular bacteria with lower *rrn* operon copies, including *Campylobacter*, *Legionella*, *Brucella*, and *Bordetella* (all of which have 3 *rrn* operon copies), *Chlamydia* and *Helicobacter* (2 copies), and *Mycoplasma*, *Coxiella*, *Rickettsia*, and *Mycobacterium avium*-*M. intracellulare* (1 single rRNA gene copy) (48, 49)? As can be seen in the example in Fig. S3, erythromycin and chloramphenicol, antibiotics that are deemed bacteriostatic for *E. coli*, are strongly bactericidal for another bacterial species, such as *Campylobacter jejuni*, which has 3 *rrn* operons rather than the 7 of *E. coli*.

10.1128/mBio.02253-16.3 FIG S3  Changes in the viable cell density of *Campylobacter jejuni* exposed to erythromycin and chloramphenicol. For this experiment, *C. jejuni* NCTC11168 was cultivated in Mueller-Hinton II (MH II) broth (Oxoid) or brain heart infusion (BHI) broth (Oxoid) at 37°C under microaerobic conditions (6% O_2_, 6% CO_2_, 88% N_2_). For MIC determination, an exponentially growing culture was diluted with MH II broth to an optical density at 600 nm (OD_600_) of 0.002 (ca. 2 × 10^6^ CFU/ml) and 0.1-ml aliquots were added to 96-well microtiter plates containing 0.1 ml 2-fold dilutions of erythromycin (0.13 to 64 μg/ml) and chloramphenicol (0.39 to 200 μg/ml) in MH II broth. Following 24 h incubation under the conditions described above, the MICs of erythromycin and chloramphenicol were assessed as 1 μg/ml and 3.1 μg/ml, respectively. For time-kill experiments, *Campylobacter* was cultivated for 4 to 6 h in BHI broth to an optical density (OD_600_) of 0.01 (ca. 10^7^ CFU/ml); 10-ml suspensions were supplemented with antibiotics and incubated at 37°C under microanerobic conditions. At indicated intervals, the viable cell density was estimated by serial dilution (0.9% saline solution) and plating on base II agar (Oxoid) supplemented with 5% bovine blood. Results are presented as means and standard deviation of the results of three independent experiments with two replicates. The error bars are smaller than the tic marks and therefore are not apparent in this figure. Download FIG S3, TIF file, 0.4 MB. Copyright © 2017 Levin et al. 2017 Levin et al. This content is distributed under the terms of the Creative Commons Attribution 4.0 International license.

Finally, we cannot discard the possibility of using bacterial mutants with low ribosome numbers in screening tests of natural or synthetic compounds to detect new ribosome-acting agents (eventually with higher cidal activity) or of studying the possibility of increasing the bactericidal effect of hitherto bacteriostatic antibiotics, eventually targeting more effectively ribosome subunit assembly (50).

### Coda: hypotheses are to be tested, modified, and expanded upon, not championed.

While there has been a great deal of impressive research on the molecular and structural biology of antibiotic action, the mechanisms by which these drugs actually kill bacteria and/or inhibit their replication remain largely unknown and subjects of controversy. It may well be that the “numbers game” hypothesis for the abatement of growth and low rate of decline in viable cell density of bacteria exposed to ribosome-targeting bacteriostatic antibiotics presented here will be modified and expanded upon by additional models and experiments. We “venture” this hypothesis because it is compelling *a priori*, because it has the virtue of parsimony, and because it is supported by the results of experiments that could have rejected it. We also see it as a path to address this issue of the mode of action of ribosome-targeting bacteriostatic antibiotics and to obtain a fully mechanistic answer (or answers).

## MATERIALS AND METHODS

### Bacteria.

For detailed information about the ribosomal operon and *rrn* deletion strains developed by S. Quan and colleagues, see Table 1. For detailed genetic information about these strains, see references 24 and 25. These strains were obtained from two sources, the *E. coli* Genetic Stock Center at Yale University and Ole Skovgaard at Roskilde University in Denmark.

**TABLE 1:** Ribosomal operon and *rrn* deletion strains developed by S. Quan and colleagues

*E. coli* strain (Quan SQ notation)	Designation in this study
SQ37	D1
SQ40	D2
SQ49	D3
SQ78	D4
SQ88	D5
SQ110	D6
MG1655 (ancestor)	MG

### Medium.

Difco Davis minimal medium Ca (casein amino acids)—designated DMCa—is composed of 1 g (NH_4_)_2_SO_4_, 3 g KH_2_PO_4_, 7 g K_2_HPO_4_, and 0.5 g sodium citrate (Na_3_C_6_H_5_O_7_).

After autoclaving, the following were added to 1 liter of that medium: 100 ml 2% casein amino acids (Difco), 1 ml 1% vitamin B1, 1 ml 1% Mg_2_SO_4_, and 1 g glucose.

### Antibiotics, sources, and MICs.

The sources and antibiotics were as follows: from Sigma, tetracycline hydrochloride (TET), chloramphenicol (CAM), and gentamicin (GEN); from AppliChem, ciprofloxacin (CIP); and from Tocris Bio-Techne, azithromycin (AZI).

The MICs of these antibiotics were estimated by the standard factor-of-2 serial dilution protocol (28) but in DMCa. The MICs are listed in Table S1 in the supplemental material for MG1655 and the *rrn* operon deletion strains.

### Sampling.

Lysogeny broth agar (Difco) was used for sampling.

### Extraction of RNA and estimation of the fraction of 16S and 23S RNA.

RNA was isolated from overnight DMCa cultures of the *rrn* operon deletion strains using a PureLink minikit and an on-column PureLink DNase treatment kit, both from Life Technologies, Inc. The relative fractions of 16S and 23S RNA in the extracted RNA were estimated with an Agilent 20100 Bioanalyzer by the Emory University Integrated Genomics Core (EIGC) facility.

### Estimates of the maximum growth rates and lags.

Overnight cultures were diluted to ~5 × 10^5^ cells per ml in DMCa. A 300-μl volume of the cell suspensions was added to the wells of 100-well Bioscreen plates. The plates were continuously shaken, and the optical densities were determined (at 600 nm) every 5 min. As noted in reference 51, using initial densities of 5 × 10^5^ CFU/ml, the turbidity of the culture at time zero was undistinguishable from that of uninoculated medium, thereby enabling us to normalize the densities by subtracting the time zero OD for each well, rather than using a blank. The exponential growth rates (the Malthusian parameters [MP]) of these cultures were estimated as the maximum slope of the natural logarithm of the optical density as a function of time. Specifically, the MP was estimated as the greatest slope calculated over five consecutive points (a time span of 25 min), after the normalized OD reached 0.02, as suggested in reference 51.

The lag is defined here as the extent of time before there is a net increase in cell density. We are assuming that, prior to that time, the viable cell density of the population was constant. And we are further assuming that, immediately following the lag, at time *t*_lag_, the population was growing at its maximum rate (*r*) per cell per hour. Thus, if *N*(0) and *N*(*t*) are the cell densities at initiation of the experiment and after *t* hours, respectively, if t* is the time required for the population to reach a threshold density N*, the lag phase can be calculated as

(1) $$t_{lag}=t*-\frac{1}{r}log\frac{N^{*}}{N(0)}$$

For our estimate of *N*(0) for this calculation, we use the CFU estimate of density of the culture at the start of the experiment, when the wells were filled. As our estimate of the threshold density, N*, we use the results of an experiment performed with MG1655 and the 6 *rrn* deletion strains growing exponentially in the experimental media in the Bioscreen. In this experiment, we estimated the CFU density of the cultures when the optical density (at 600 nm) in wells of the Bioscreen was 0.02. As our estimate of the CFU densities (N*) of the cultures at an optical density of 0.02, we use the mean CFU estimate of the densities of all 7 cultures at an optical density of 0.02, or approximately 10^7^ cells per ml. For example, if a culture was growing at a rate of *r* = 0.025 per cell min, if *N*(0) = 2 × 10^5^ cells per ml, and if an OD (600 nm) of 0.02 (a CFU density of 10^7^ cells per ml) was reached at t* = 250 min after the start of the experiment, from equation 1, the estimated lag (*t*_lag_) would be 93.5 min.

### Time-kill experiments and estimating growth rates/death rates (ψ).

The bacteria were grown overnight in DMCa. These cultures were diluted in this media and allowed to grow for 2.5 h, at which time 2 ml or 2.5 ml of the cultures was placed into the wells of 12-well or 24-well Falcon polystyrene plates. The antibiotics were added at the noted concentrations. These plates were incubated with shaking. At defined intervals, 100-µl samples were taken and viable cell densities were estimated from CFU data by diluting and plating the samples on LB agar.

For the bacteriostatic drugs (Fig. 3), samples were taken at time zero and at 24 h and three independent estimates of the viable cell density were made for each sample. For our estimates of the rates of growth and antibiotic-mediated killing by the bactericidal drugs (Fig. 4) samples were taken at relatively short intervals that differed among the drugs, 0.75 and 1.5 h for ciprofloxacin and 0.5 and 1 h for the gentamicin. The hourly rates of growth/death of these cultures (ψ), calculated from the viable cell densities (CFU data) of growing cultures immediately before exposure to the antibiotics, *N*(0), and after 24 h of exposure, *N*(24), were determined as follows: $\psi_{i}\;=\;\frac{1}{24}\;\;\ln\;\;\left[\frac{N(24)}{N(0)}\right]$. The estimates of *N*(0) and *N*(24) for these calculations were the mean values estimated from three (and occasionally two) separate serial dilutions and platings.

### Post-antibiotic effects.

Fresh overnight cultures of the strains were diluted 1/100 in fresh DMCa and incubated at ~2 h and 37°C with shaking. At that time, four cultures were prepared for each strain, one each with azithromycin, chloramphenicol, or tetracycline at 40, 125, or 25 µg/ml, respectively, and one as an antibiotic-free control. After 3 h of incubation at 37°C with shaking, all the cultures were plated in triplicate to estimate the viable cell (CFU) densities. For each antibiotic and the controls, three sets of cultures were prepared to measure their growth dynamics in a Bioscreen using five wells for each of the cultures. (i) For the washed antibiotic-exposed cells, the antibiotic-exposed cultures were diluted 1/1,000 in fresh media, which brought the antibiotic concentration to about 0.005× the azithromycin, chloramphenicol, or tetracycline MIC (0.04, 0.125, or 0.025 µg/ml, respectively) (antibiotic-exposed cultures). (ii) For the antibiotic controls, unexposed cells were diluted 1/10,000 in fresh media with 0.005× the MIC of the antibiotics. These antibiotic controls are employed to determine the effects on lag of the residual drugs in the washed antibiotic-exposed cultures. (iii) For the controls, unexposed cells were diluted 1/10,000, 1/100,000, or 1/1,000,000 in fresh media to cover the range of cell densities in the washed antibiotic-exposed cultures.

Using the above-described method, we estimated the lag of (i) the washed antibiotic-exposed cells in fresh medium (LagW), (ii) the control cultures with 0.005× MIC of the antibiotic (LagCA), and (iii) the antibiotic-free control cultures (LagC). The post-antibiotic effect (PAE) is defined as the difference between LagW and the largest value among the LagCA and LagC results. This gives a conservative estimate of lag. Save for the method employed to estimate the lag, which controls for possible differences in growth rates, the procedure to estimate the PAE in these experiments is similar to that described in reference 52.
