# Supplementary figures and images for: A Numbers Game: Ribosome Densities, Bacterial Growth, and Antibiotic-Mediated Stasis and Death
Source: mBio. 2017 Feb 7;8(1):e02253-16. doi: 10.1128/mBio.02253-16 (PMC5296603; doi:10.1128/mBio.02253-16)

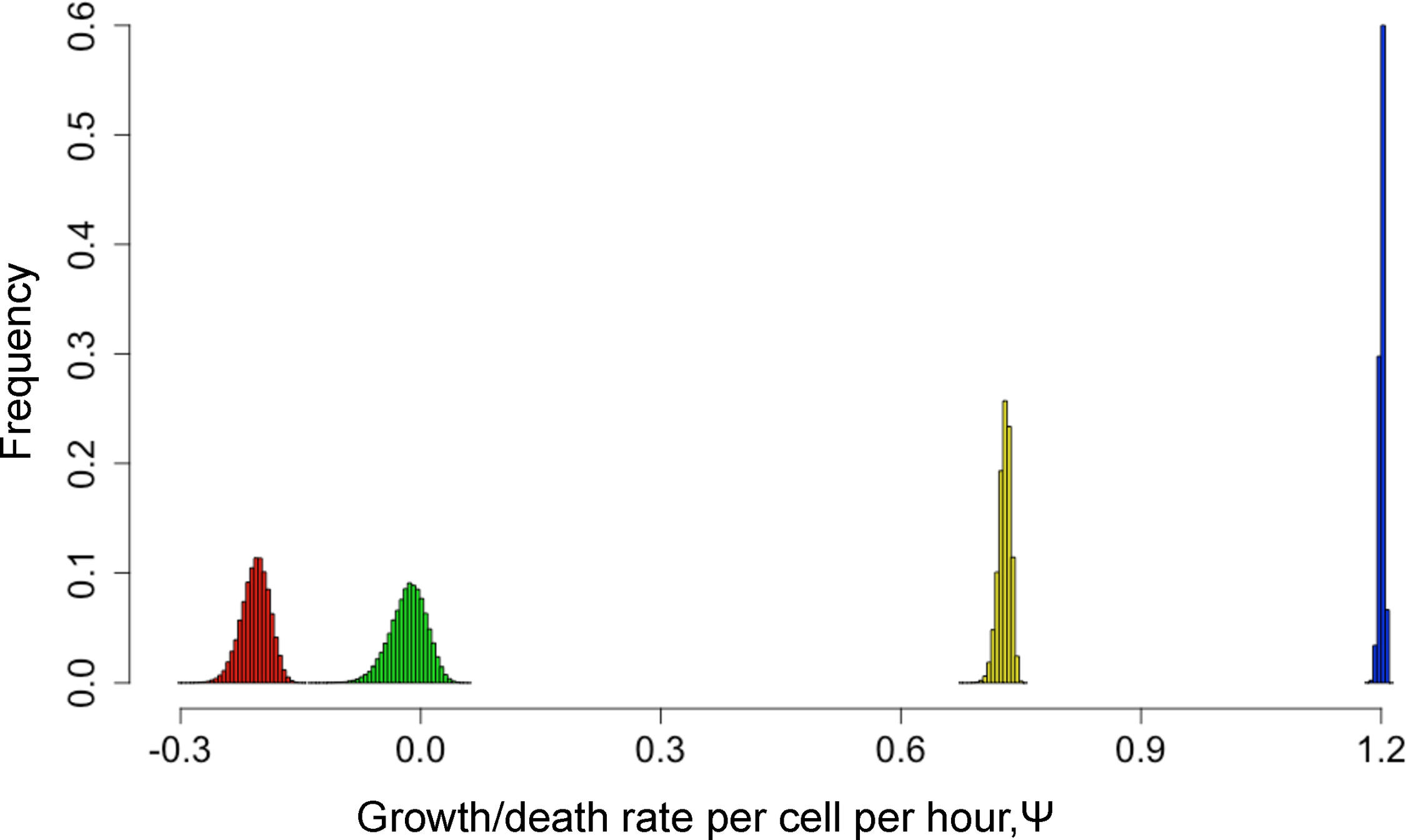

Supplement: FIG S1 [file mbo001173176sf1.tif]

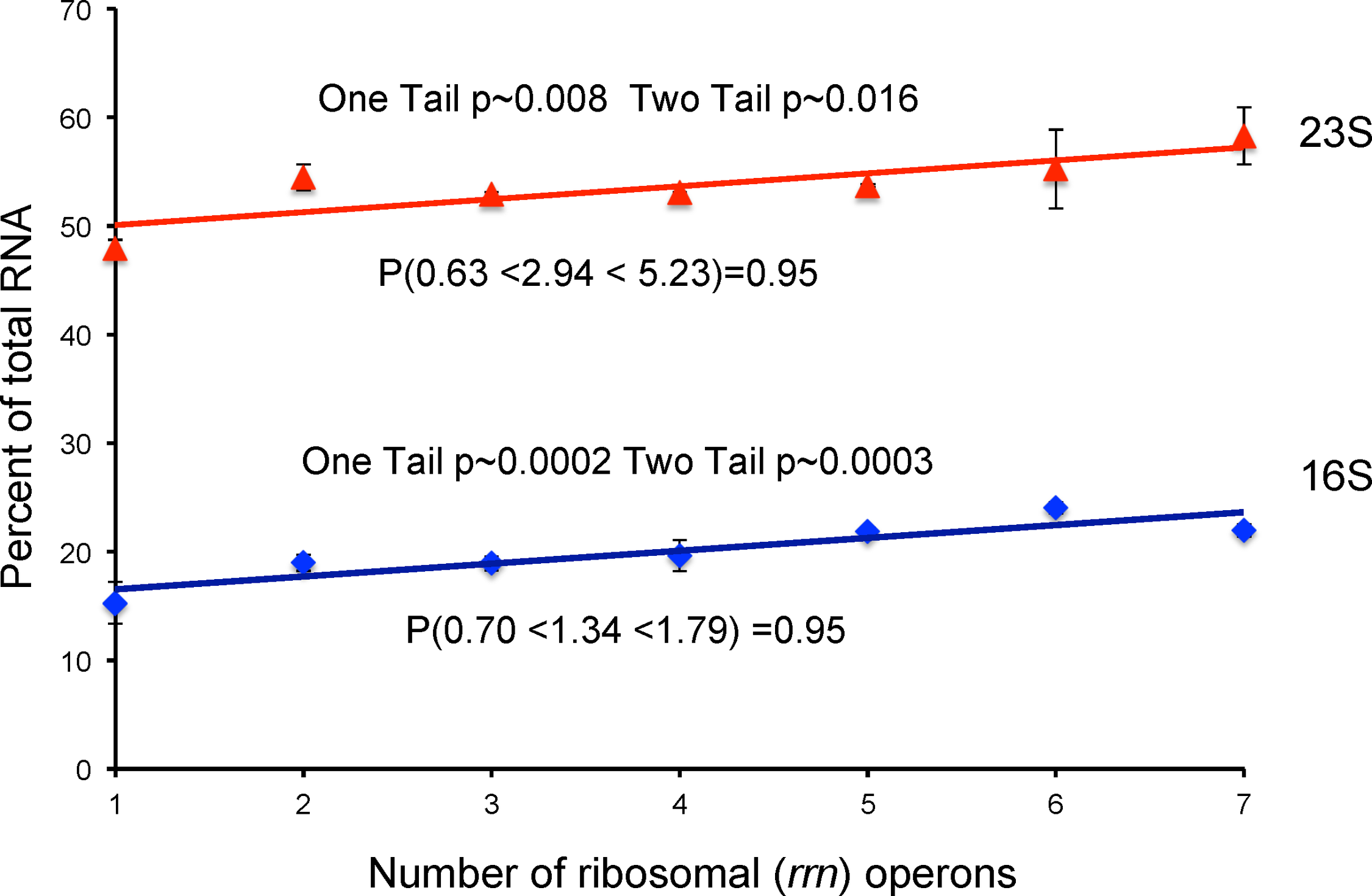

Supplement: FIG S2 [file mbo001173176sf2.tif]

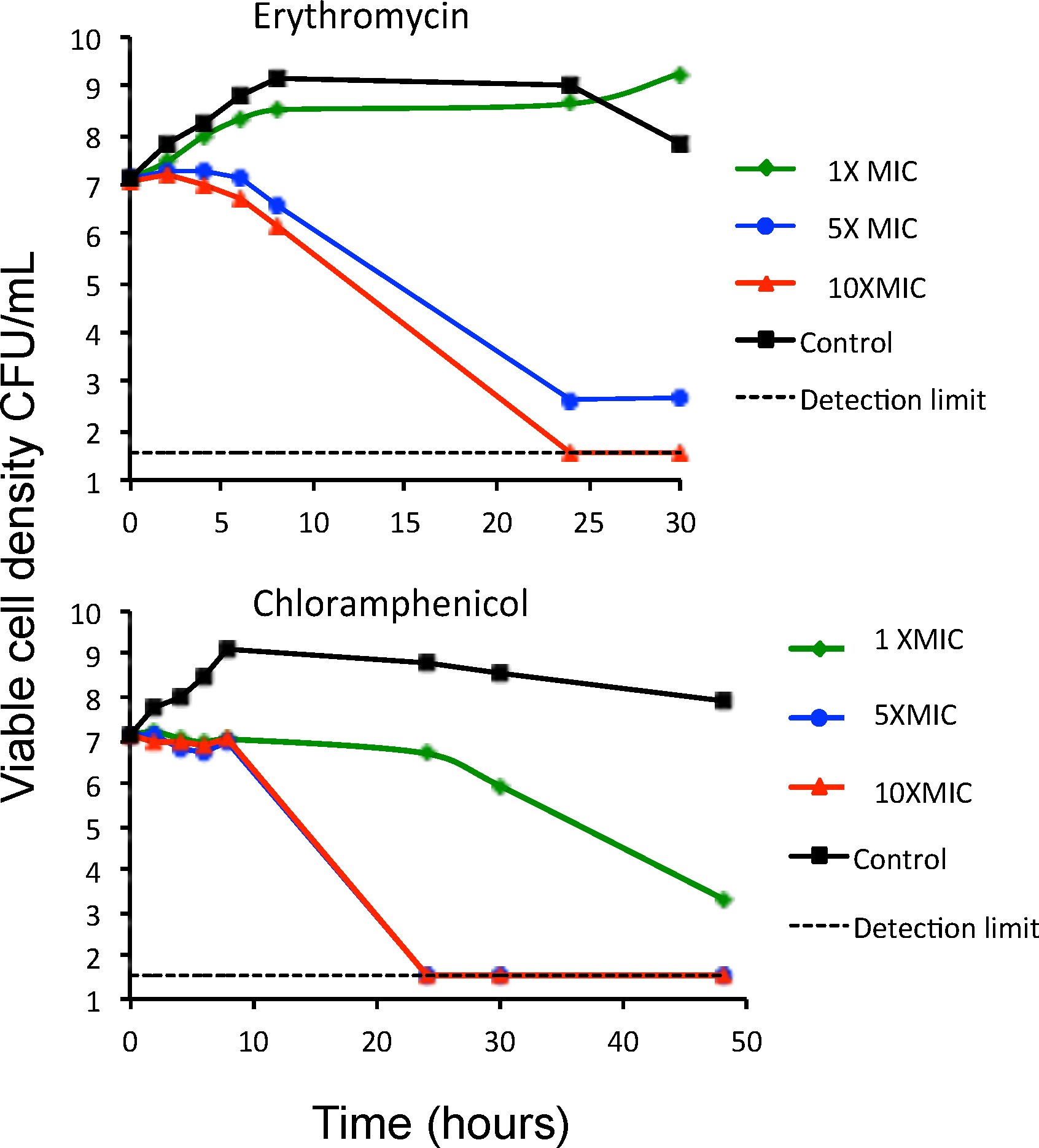

Supplement: FIG S3 [file mbo001173176sf3.tif]
