# Supplementary material for: A Numbers Game: Ribosome Densities, Bacterial Growth, and Antibiotic-Mediated Stasis and Death
Source: mBio. 2017 Feb 7;8(1):e02253-16. doi: 10.1128/mBio.02253-16 (PMC5296603; doi:10.1128/mBio.02253-16)
Supplement: TABLE S1 [file mbo001173176st1.docx]

| **MIC** | **MG** | **D1** | **D2** | **D3** | **D4** | **D5** | **D6** |
| --- | --- | --- | --- | --- | --- | --- | --- |
| GEN | 2.5 | 2.50 | 2.50 | 2.50 | 2.50 | 1.25 | 0.63 |
| TET | 0.63 | 1.25 | 0.63 | 0.63 | 0.63 | 0.63 | 0.63 |
| AZI | 1.25 | 1.25 | 1.25 | 2.50 | 1.25 | 2.50 | 2.50 |
| CAM | 3.75 | 7.50 | 3.75 | 3.75 | 3.75 | 3.75 | 3.75 |
| CIP | 0.125 | 0.13 | 0.06 | 0.25 | 0.06 | 0.13 | 0.13 |
